# Comparison between central and ambulatory blood pressure measurements in early detection of end organ damage: a single-center prospective non-randomized controlled trial

**DOI:** 10.1186/s43044-019-0013-3

**Published:** 2019-09-10

**Authors:** Doaa A. Fouad, Hosam Hassan Al Araby, Mohammad Ashraf, Ahmed El-Sherif El-Kousy

**Affiliations:** Department of Cardiology, Faculty of Medicine, Assiut University Hospital, Assiut, Egypt

## Abstract

**Background:**

Both ambulatory blood pressure (AMBP) and non-invasive central blood pressure (NCBP) monitoring could be used as predictors for early detection of hypertensive end organ damage (EOD). However, the comparison between these two methods needs more clarification. Our cross-sectional study included 100 hypertensive patients with a mean age of 47.52 ± 8.35 years on regular antihypertensive treatment for ≥ 1 year (50 controlled, 50 uncontrolled). We compared associations, sensitivity, and specificity of EOD parameters with office, AMBP, and NCBP measurements. We measured left ventricular mass index (LVMI), carotid intimal medial thickness (CIMT), ankle-brachial index (ABI), serum creatinine, glomerular filtration rate (GFR), and pulse wave velocity (PWV).

**Results:**

We found a significant relation between SBP of NCBP, AMBP and LVMI, and CIMT, PWV, and GFR respectively (*P* < 0.05) while office SBP showed no significant relation. Systolic AMBP showed a high sensitivity to ABI (98%) and CIMT (92%) while systolic NCBP had 92% specificity and DBP showed 90% sensitivity for ABI.

**Conclusion:**

AMBP and NCBP show a significant relation to LVMI, CIMT, PWV, and GFR with little superiority of central BP while office BP does not. Systolic ABPM has high sensitivity to ABI and CIMT and systolic NCBP has a high sensitivity and specificity to ABI.

## Background

Reliability of brachial blood pressure (BP) measurement in the physician’s office has its own limitations, while out-of-office BP measurement, using either home BP monitoring or ambulatory BP monitoring (ABPM) techniques, is devoid of such limitations and is gaining importance in the management of hypertension.

It is now generally accepted that ABPM is an important adjunct to conventional office BP measurement, and ABPM became the “gold standard” for screening, diagnosis, and management of hypertension against the important limitations of office BP which have led to the increasingly frequent suggestion that ABPM [1]. Among the numerous benefits of ABPM, the avoidance of potential BP measurement errors such as observer bias, terminal digit preference, and provision of more comprehensive information on BP behavior is possible with office or home BP. Ambulatory blood pressure monitors overcome this problem by obtaining multiple readings over the 24-h period and capturing the blood pressure variability [2].

Current non-invasive strategies for evaluating central aortic BP require recording of a blood vessel weight wave utilizing the MOBIL-O-GRAPH BP device which utilizes brachial BP waves for a non-invasive estimation of central BP [3]. When brachial artery blood pressure was used to stratify blood pressure measurements, a considerable overlap in aortic systolic pressure was observed, such that over 70% of individuals were categorized as having “high-normal” brachial systolic pressure based on Joint European Cardiology and Hypertension Society guidelines [4], and the authors postulated that we may be treating some patients with relatively low central pressures and not treating individuals with elevated central pressures, because they have brachial systolic pressures under current treatment thresholds. McEniery et al. [5] suggested that NICBP will have important clinical implications if central pressure turns out to be a better predictor of cardiovascular risk, because it suggests that. The Dicomano Study in Italy [6] and a Taiwanese study [7] also observed a stronger association between cardiovascular events and central, rather than brachial pressure. The heart, kidneys, and major arteries supplying the brain are exposed to aortic rather than brachial pressure. Therefore, there is a strong rationale to believe that cardiovascular events may ultimately be more closely related to central rather than brachial pressure. Evidence published over the last 12 years is concerning the relationship between central pressures [8].

## Methods

The study group included 100 hypertensive patients recruited from the hypertension clinic in Assiut University Hospital, during the period from June 2016 to July 2017. Patients were divided into two groups, either controlled or uncontrolled office BP according to the European Society of Cardiology (ESC) guidelines 2013 (≥ 140 mmHg systolic BP (SBP) and/or ≥ 90 mmHg diastolic BP (DBP).

A detailed history was obtained, and complete clinical examination was performed including patients’ weight and height to calculate body mass index (BMI).

Office pressure blood pressure measurement was done according to ESC 2013; we measured the systolic and diastolic blood pressure and compared the results with those of ABPM and NICBP.

For measurements of ABPM, we used NICE recommendations to ensure that at least two measurements per hour are taken during the person’s usual waking hours. We used the average value of at least 14 measurements taken during the person's usual waking hours to confirm a diagnosis of hypertension. The device was programmed to obtain BP readings at 30-min interval during the day (08:00–22:00 h) and at 60-min intervals during the night (22:00–08:00 h) [9]. We measured systolic and diastolic blood pressure and compared the results with those of office BP and NICBP.

Blood pressure varies over 24 h with a number of well-recognized patterns. Dippers are individuals characterized by at least a 10% decline in nocturnal BP compared to their awake BP [10]. Most of the patients are dippers. Non-dippers are individuals having blunted or absent blood pressures which decline during sleep [11]. Approximately 10–30% of patients are “non-dippers,” and in our study, non-dippers represent 34% of our patients.

We obtained the central blood pressure curve of our patients in a quiet, temperature-controlled examination room at the hypertension clinic. Three measurements were taken with a 2-min break between them by using Mobilograph device. The procedure was performed with the patient in a supine position and using an adequately sized cuff (the Mobil-O-Graph 24 h PWA ABPM device (IEM, Stolberg, Germany) [12, 13]).

We measured systolic and diastolic blood pressures and compared them with the measurements of both office and ABPM.

We measured pulse wave velocity (m/s) (PWV) by Mobilograph and compared the result and age to detect the degree of arterial thickness related to hypertension. The slower the pulse wave velocity is, the better the heart health is. However, normal pulse wave velocity values vary according to age. We measured also the mean arterial pressure (MAP) and augmentation index (AI).

Blood samples were collected to measure serum creatinine and to exclude patients with a glomerular filtration rate of < 30 ml/min/1.73 m^2^. The measurement of GFR was obtained by the Cockcroft-Gault formula study equation.

ECG was used to measure the left ventricular hypertrophy (LVH) using “Sokolow-Lyon ≥ 38 mm [14]. Echocardiography using Phillips IE33 ultrasound system was performed to all patients in addition to controls. Images were obtained from the short-axis view and longitudinal parasternal 4-chamber, 2-chamber, and 5-chamber slices. LV mass (LVM) and LVMI were calculate using LVMI = LV mass/BSA calculated by the Mosteller formula [15]. Ejection fraction (EF %) was calculated using Simpson’s method [16].

Carotid intimal medial thickness (CIMT) was measured with Doppler ultrasound (US) on the carotid artery to detect increased thickness related to hypertension. The intima-media complex can easily be distinguished from the surrounding tissue in US images, and the distinct borders allow for manual as well as automatic measurements of the CIMT. Damage is defined as the presence of CIMT > 0.9 mm or plaque [17].

ABI was calculated by measuring the systolic blood pressure from both brachial arteries and from both the dorsalis pedis and posterior tibial arteries at rest in the supine position. The systolic pressures were recorded with a handheld 5- or 10-mHz Doppler instrument [18, 19]. Normal ABI ranges from 1.0 to 1.4; values above 1.4 suggest a non-compressible calcified vessel. However, a value below 0.9 is considered diagnostic of peripheral arterial disease (PAD).

## Statistical analysis

The data were tested for normality using the Anderson-Darling test and for homogeneity variances prior to the further statistical analysis. Categorical variables were described by the number and percent (*N*, %), where continuous variables are described by mean and standard deviation (mean, SD). The chi-square test and Fisher exact test were used to compare categorical variables where comparisons between continuous variables were done by *t* test and independent samples *t* test ANOVA. A two-tailed *p* < 0.05 was considered statistically significant. We used the Pearson and Spearman correlation to determine the association between variables. All analyses were performed with the IBM SPSS 20.0 software. The receiver operating characteristic (ROC) curve was done for the most powerful predictors for determination of cutoff point to be a risk factor for end organ damage. Post hoc tests are run to confirm where the differences occurred between groups; they should only be run when you have a shown an overall statistically significant difference in group means (i.e., a statistically significant one-way ANOVA result).

## Results

**Fig. 1:**
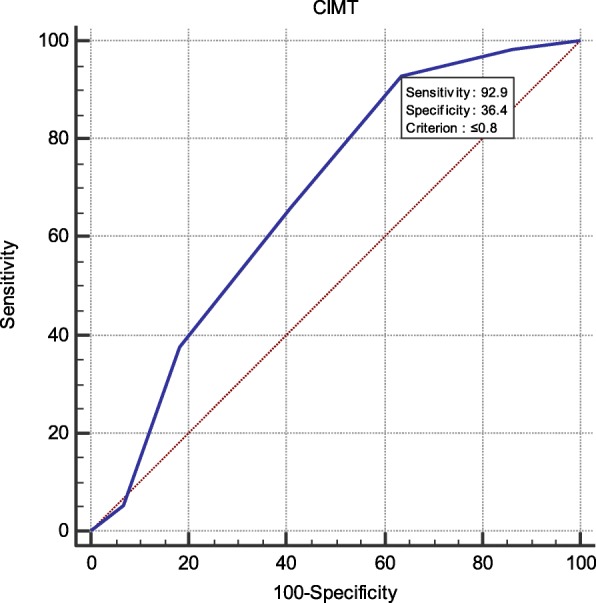
High sensitivity of ABPM DBP with CIMT (92.9%)

**Fig. 2:**
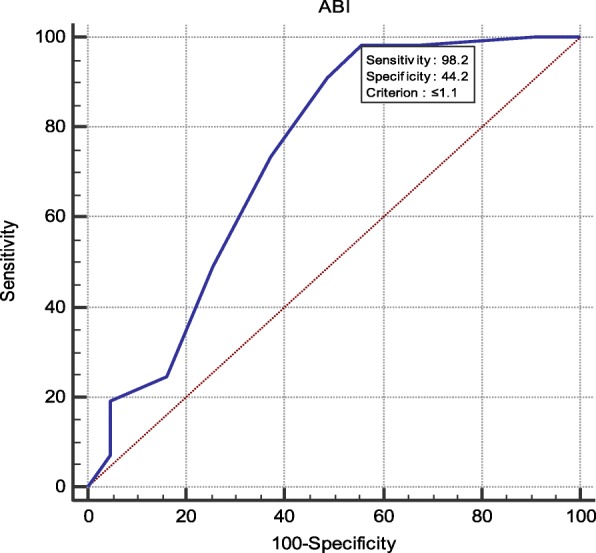
High sensitivity of ABPM SBP with ABI (98.2%)

**Fig. 3:**
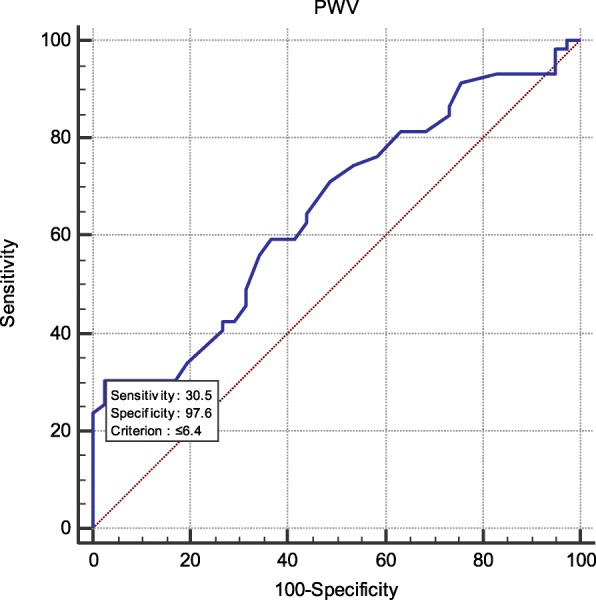
High specificity of central SBP with PWV (97.6%)

**Fig. 4:**
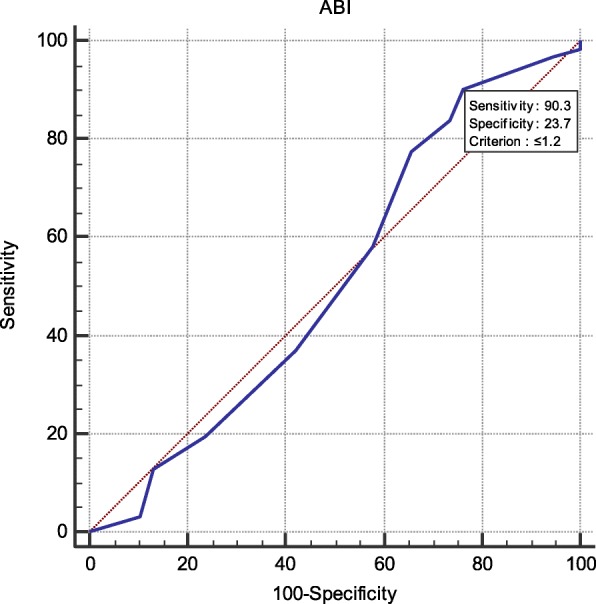
High sensitivity of central DBP with ABI (90.3%)

The demographic and clinical characteristics of the study population are presented in Tables 1, 2, 3, 4 and 5, (Figs. 1, 2, 3 and 4).

**Table 1:** Characteristics of the controlled versus uncontrolled groups with office blood pressure measurement

Variable	Controlled, *N* = 50	Uncontrolled, *N* = 50	*P*	All, *N* = 100
Male	12 (24%)	15 (30%)	0.499	27 (27%)
Female	38 (76%)	35 (70%)		73 (73%)
Age	48.1 ± 7.75	46.94 ± 8.96	0.490	47.52 ± 8.35
BMI	32.7 ± 5.6	31 ± 5.22	0.119	31.85 ± 5.45
Smoking	12 (24%)	13 (26%)	0.817	25 (25%)
Duration of HTN	6.78 ± 4.47	6.1 ± 3.73	0.411	6.44 ± 4.11
F. history	28 (56%)	27 (54%)	0.841	55 (55%)
Office systolic	135.12 ± 19.45	143.56 ± 29.4	0.087	138.58 ± 24.25
Office diastolic	88.53 ± 16.65	85.37 ± 15.04	0.341	87.33 ± 16.05
ABPM systolic	125.15 ± 17.93	128.96 ± 25.9	0.395	126.05 ± 20.42
ABPM diastolic	87.63 ± 18.52	86.04 ± 12.47	0.616	86.84 ± 15.99
Night				
Dipper	36 (72%)	30 (60%)	0.205	66 (66%)
Not dipper	14 (28%)	20 (40%)		34 (34%)
Central systolic	126.07 ± 19.41	130.63 ± 27.61	0.348	127.44 ± 22.14
Central diastolic	89.49 ± 17.93	88.77 ± 13.89	0.831	89.21 ± 16.40
PWV	7.25 ± 1.09	7.8 ± 1.33	0.024*	7.52 ± 1.24
Augmentation index	24.3 ± 9.71	25.84 ± 11.75	0.477	25.07 ± 10.75
MAP	102.42 ± 16.27	98.88 ± 14.53	0.254	100.65 ± 15.45
Cr	0.8 ± 0.31	1.04 ± 0.34	0.000**	0.92 ± 0.35
GFR	118.04 ± 48.29	94.88 ± 51.17	0.022*	106.46 ± 50.85
LVMI	0.92 ± 0.12	0.95 ± 0.13	0.311	0.93 ± 0.13
EF	59.34% ± 5.69%	59.12% ± 5.83%	0.849	59.23% ± 5.73%
CIMT	0.7 ± 0.12	0.77 ± 0.14	0.008**	0.74 ± 0.13
ABI	0.91 ± 0.2	0.95 ± 0.27	0.322	0.93 ± 0.24

*BMI* body mass index, *HTN* hypertension, ABPM ambulatory blood pressure monitoring, *PWV* pulse wave velocity, *MAP* mean arterial pressure, *Cr* creatinine, *GFR* glomerular filtration ratio, *LVMI* left ventricular mass index, *EF* ejection fraction, *CIMT* carotid intimal thickness, *ABI* ankle brachial index

* Statistically significant difference (*p* < 0.05), **highly statistically significant difference (*p* < 0.01)

**Table 2:** Blood pressure variability

	ABPM SBP “controlled”	Central SBP “controlled”	*P*
SBP	57 (57%)	70 (70%)	0.056
DBP	56 (56%)	61 (61%)	0.473
Mean BP	57 (57%)	67 (67%)	0.419

Post hoc (LSD test), P: Comparison between ABPM SBP and central SBP

*HTN* hypertension, *ABPM* ambulatory blood pressure monitoring, *PWV* pulse wave velocity, *Cr* creatinine, *GFR* glomerular filtration ratio, *LVMI* left ventricular mass index, *CIMT* carotid intimal medial thickness, *ABI* ankle-brachial index

*Statistically significant difference (*p* < 0.05)

**Highly statistically significant difference

**Table 3:** Relation between SBP measured by different tools monitoring and early hypertensive end organ damage

	ABPM		*P* value	Central		*P* value
	Controlled	Not controlled		Controlled	Not controlled	
	No. (%)	No. (%)		No. (%)	No. (%)	
ECG						
Normal	48 (96%)	13 (26%)	0.000**	49 (90.7%)	12 (26.1%)	0.000**
LVH	2 (4%)	34 (68%)		5 (9.3%)	31 (67.4%)	
LBBB	0 (0%)	3 (6%)		0 (0%)	3 (6.5%)	
LVMI						
Normal	47 (94%)	37 (74%)	0.006**	52 (96.3%)	32 (69.6%)	0.000**
Abnormal	3 (6%)	13 (26%)		2 (3.7%)	14 (30.4%)	
CIMT						
Normal	47 (94%)	37 (74%)	0.006**	51 (94. 4%)	33 (71.7%)	0.002**
Abnormal	3 (6%)	13 (26%)		3 (5. 6%)	13 (28.3%)	
PWV						
Normal	50 (100%)	43 (86%)	0.006**	54 (100%)	39 (84.8%)	0.003**
Abnormal	0 (0%)	7 (14%)		0 (0%)	7 (15. 2%)	
ABI						
Normal	39 (78%)	21 (42%)	0.000**	41 (75.9%)	19 (41.3%)	0.000**
Abnormal	11 (22%)	29 (58%)		13 (24.1%)	27 (58.7%)	
Creatinine						
Normal	47 (94%)	30 (60%)	0.000**	52 (96.3%)	25 (54.3%)	0.000**
Abnormal	3 (6%)	20 (40%)		2 (3.7%)	21 (45.7%)	
GFR						
Normal	47 (94%)	36 (72%)	0.003**	50 (92.6%)	33 (71.7%)	0.006**
Abnormal	3 (6%)	14 (28%)		4 (7.4%)	13 (28.3%)	

Chi-square test

*Statistically significant difference (*p* < 0.05)

**Highly statistically significant difference (*p* < 0.01)

*HTN* hypertension, *ABPM* ambulatory blood pressure monitoring, *PWV* pulse wave velocity, *Cr* creatinine, *GFR* glomerular filtration ratio, *LVMI* left ventricular mass index, *CIMT* carotid intimal thickness, *ABI* ankle-brachial index

**Table 4:** The correlation between each blood pressure monitoring and hypertensive end organ damage parameters

		LVMI	CIMT	PWV	ABI	Cr	GFR	ECG
ABPM SBP	*R*	0.326	0.385	0.336	0.439	0.473	− 0.265	0.793
	*P*	< 0.001**	< 0.001**	< 0.001**	< 0.001**	< 0.001**	0.008*	< 0.001**
ABPM DBP	*R*	0.286	0.317	0.214	0.418	0.325	− 0.181	0.656
	*P*	0.004**	< 0.001**	0.032	< 0.001**	< 0.001**	0.072	< 0.001**
Central SBP	*R*	0.347	0.330	0.369	0.437	0.439	− 0.254	0.720
	*P*	< 0.001**	< 0.001**	<0.001**	< 0.001**	< 0.001**	0.011*	< 0.001**
Central DBP	*R*	0.111	0.118	0.209	0.156	0.133	0.046	0.506
	*P*	0.271	0.243	0.037*	0.120	0.186	0.651	< 0.001**

*R* value is the correlation coefficient r measures the strength and direction of a linear relationship between two variables on a scatterplot. The value of r is always between + 1 and − 1

*SBP* systolic blood pressure, *DBP* diastolic blood pressure, *HTN* hypertension, *ABPM* ambulatory blood pressure monitoring, *PWV* pulse wave velocity, *Cr* creatinine, *GFR* glomerular filtration ratio, *LVMI* left ventricular mass index, *CIMT* carotid intimal thickness, *ABI* ankle-brachial index

*Statistically significant difference (*p* < 0.05)

**Highly statistically significant difference (*p* < 0.01)

**Table 5:** Logistic regression models for hypertensive end organ damage

	ECG				LVMI			
	Odds ratio	Confidence interval		*P* value	Odds ratio	Confidence interval	*P* value	
AMBP SBP (uncontrolled/controlled)	0.081	0.029–0.224		0.000**	0.200	0.065–0.617	0.005**	
AMBP MAP (uncontrolled/controlled)	0.101	0.030–0.334		0.000**	0.167	0.053–0.529	0.002**	
Central SBP (uncontrolled/controlled)	0.670	0.271–1.656		*0.386*	0.376	0.124–1.143	*0.085*	
Central MAP (uncontrolled/controlled)	0.286	0.117–0.697		0.006**	0.355	0.119–1.060	*0.063*	
	CIMT				PWV			
	Odds ratio	Confidence interval		*P* value	Odds ratio	Confidence interval	*P* value	
AMBP SBP (uncontrolled/controlled)	0.514	0.172–1.538		*0.234*	0.061	0.007–0.535	0.012*	
AMBP MAP (uncontrolled/controlled)	0.706	0.201–2.478		*0.587*	0.298	0.061–1.458	*0.135*	
Central SBP (uncontrolled/controlled)	0.059	0.006–0.524		0.014*	0.065	0.008–0.564	0.010*	
Central MAP (uncontrolled/controlled)	0.355	0.119–1.060		*0.063*	0.075	0.010–0.602	0.014*	
ABI			Cr			GFR		
Odds ratio	Confidence interval	*P* value	Odds ratio	Confidence interval	*P* value	Odds ratio	Confidence interval	*P* value
0.144	0.056–0.371	0.000**	0.383	0.146–1.003	*0.051*	0.487	0.171–1.388	*0.178*
0.206	0.071–0.598	0.004**	0.259	0.090–0.743	0.012*	0.412	0.142–1.283	*0.126*
0.710	0.288–1.752	*0.457*	1.350	0.445–4.098	*0.596*	0.474	0.161–1.392	*0.174*
0.556	0.234–1.322	*0.184*	2.192	0.832–5.778	*0.112*	1.140	0.367–3.543	*0.820*

Logistic regression included the following independent variables: central SBP: uncontrolled = 1/controlled = 2; central MAP: abnormal = 1/normal = 2; ABPM SBP: uncontrolled = 1/controlled = 2; ABPM MAP: uncontrolled = 1/controlled = 2. Values in italics represent significant *P* value < 0.05

*ABPM* ambulatory blood pressure monitoring, *PWV* pulse wave velocity, *Cr* creatinine, *GFR* glomerular filtration ratio, *LVMI* left ventricular mass index, *CIMT* carotid intimal thickness, ABI ankle-brachial index

*Statistically significant difference (*p* < 0.05)

**Highly statistically significant difference (*p* < 0.01)

## Discussion

In our study, we found that 24 h ABPM and non-invasive central BP monitoring are good predictable measures for early detection of hypertensive end organ damage.

In accordance with our results, Hansen et al. compare between 24 h ABPM and office BP. ABPM and office BP were entered in the same multivariate models, only the ABPM was a significant predictor of all-cause mortality and cardiovascular mortality; however, the relative risks of cardiovascular mortality were lower for office blood pressure, and office blood pressure did not predict all-cause mortality [20]. Wang et al. showed that central BP is more valuable than other blood pressure variables in predicting cardiovascular mortality [7].

After the analysis of our data, we found that all tools of BP measurements (Office –ABPM – Central) are correlated significantly with ECG changes (LVH or LBBB). ABPM SBP and DBP parameters are significantly correlated with ECG changes caused by hypertension. Central SBP, DBP, and PWV parameters are significantly correlated with ECG changes caused by hypertension.

We found that 24 h APBM and central BP are strong predictors for increasing in LVM as assessed by echocardiography. de Luca et al. showed the superiority of central SBP over brachial SBP as a major determinant of LVM regression which has recently been shown in 52 hypertensive patients treated with low-dose perindopril/indapamide and atenolol [21].

Central BP is more closely correlated with widely accepted surrogate measures of cardiovascular risk such as LVMI; a closer association of central pressure with LVMI was also found in the population-based Strong Heart Study [22] in which central systolic BP was more strongly associated with LVH determined by echocardiography. ABPM SBP and DBP parameters are significantly correlated with LVMI changes caused by hypertension. Central SBP and PWV parameters are significantly correlated with LVMI changes caused by hypertension.

Central BP and 24 h ABPM are significantly related to the changes in CIMT which are caused by hypertension. Mancia et al. had found that the end organ damage was determined not only by the degree of the high BP level but also by the 24 h ABPM. In our study, we found that both ABPM and central BP are also good predictors for CIMT changes but office BP is not conclusive for hypertensive CIMT changes. ABPM SBP and DBP parameters are significantly correlated with CIMT changes caused by hypertension. Central SBP and PWV parameters are significantly correlated with CIMT changes caused by hypertension.

PWV is an important marker for cardiovascular changes, and in our study, we find that both ABPM and central BP are significantly related to PWV with some superiority to central BP. In accordance with our results, Spronck et al. stated that office and out-of-office BP are independent predictors of aortic PWV in hypertension.

Elevated BP values over the 24 h are associated with increased aortic stiffness, so ABPM is a good predictor [23]. ABPM SBP and DBP parameters are significantly correlated with PWV changes caused by hypertension. Central SBP parameter is significantly correlated with PWV changes caused by hypertension.

In our study, we found that both 24 h ABPM and central BP are more predictable than office BP; however, Wittke et al. showed that the variability of systolic blood pressure over time derived from ABPM is associated with the ankle-brachial index [24]. ABPM SBP and DBP parameters are significantly correlated with ABI changes caused by hypertension. Central SBP, DBP, and PWV parameters are significantly correlated with ABI changes caused by hypertension.

Both ABPM and Central BP are correlated with changes in Cr level and GFR caused by hypertension in our study. However, this point is controversial with many studies, such as the Framingham Heart Study, which failed to identify an association between central BP and renal function [25] while Karras et al. [26] demonstrated that central hemodynamic and arterial stiffness parameters are associated with renal impairment. ABPM SBP and DBP parameters are significantly correlated with Cr level changes caused by hypertension. Central SBP, DBP, and PWV parameters are significantly correlated with Cr level changes caused by hypertension.

## Conclusion

AMBP and NCBP show a significant relation to LVMI, CIMT, PWV, and GFR with little superiority of central BP while office BP does not. Systolic AMBP has high sensitivity to ABI and CIMT and systolic NCBP has a high sensitivity and specificity to ABI.

## Data Availability

The data and materials are available in our cardiology department.

## Abbreviations

ABI: Ankle-brachial index
ABPM: Ambulatory blood pressure monitoring
BP: Blood pressure
BPV: Blood pressure variability
CIMT: Carotid intimal medial thickness
Cr: Creatinine
CV: Cardiovascular
DBP: Diastolic BP
ECG: Electrocardiogram
ESC: European Society of Cardiology
GFR: Glomerular filtration ratio
IMT: Intimal medial thickness
LV: Left ventricular
LVH: Left ventricular hypertrophy
LVMI: Left ventricular mass index
PWV: Pulse wave velocity
SBP: Systolic blood pressure

## References

[1] O’brien E, Parati G, Stergiou G, Asmar R, Beilin L, Bilo G (2013). European Society of Hypertension position paper on ambulatory blood pressure monitoring. J Hypertens.

[2] Anwar YA, White WB. Ambulatory monitoring of blood pressure. Blood pressure monitoring in cardiovascular medicine and therapeutics: Springer; 2001. p. 57-75.

[3] Lim SS, Vos T, Flaxman AD, Danaei G, Shibuya K, Adair-Rohani H (2012). A comparative risk assessment of burden of disease and injury attributable to 67 risk factors and risk factor clusters in 21 regions, 1990–2010: a systematic analysis for the Global Burden of Disease Study 2010. The lancet..

[4] Mancia G, Laurent S, Agabiti-Rosei E, Ambrosioni E, Burnier M, Caulfield MJ (2009). Reappraisal of European guidelines on hypertension management: a European Society of Hypertension Task Force document. Blood pressure..

[5] McEniery CM, Cockcroft JR, Roman MJ, Franklin SS, Wilkinson IB (2014). Central blood pressure: current evidence and clinical importance. European heart journal..

[6] Pini R, Cavallini MC, Palmieri V, Marchionni N, Di Bari M, Devereux RB (2008). Central but not brachial blood pressure predicts cardiovascular events in an unselected geriatric population: the ICARe Dicomano Study. J Am Coll Cardiol.

[7] Wang K-L, Cheng H-M, Chuang S-Y, Spurgeon HA, Ting C-T, Lakatta EG (2009). Central or peripheral systolic or pulse pressure: which best relates to target-organs and future mortality. Journal of hypertension..

[8] Laurent S, Boutouyrie P (2007). Arterial stiffness: a new surrogate end point for cardiovascular disease?. J Nephrol.

[9] McCormack T, Krause T, O'Flynn N (2012). Management of hypertension in adults in primary care: NICE guideline. Br J Gen Pract..

[10] Snyder F, Hobson JA, Morrison DF, Goldfrank F (1964). Changes in respiration, heart rate, and systolic blood pressure in human sleep. Journal of Applied Physiology..

[11] Staessen JA, Thijs L, Fagard R, O'brien ET, Clement D, de Leeuw PW (1999). Predicting cardiovascular risk using conventional vs ambulatory blood pressure in older patients with systolic hypertension. Jama..

[12] Jones CR, Taylor K, Chowienczyk P, Poston L, Shennan AH (2000). A validation of the Mobil O Graph (version 12) ambulatory blood pressure monitor. Blood pressure monitoring..

[13] Franssen PM, Imholz BP (2010). Evaluation of the Mobil-O-Graph new generation ABPM device using the ESH criteria. Blood Press Monit.

[14] Okin PM, Roman MJ, Devereux RB, Pickering TG, Borer JS, Kligfield P (1998). Time-voltage QRS area of the 12-lead electrocardiogram. Hypertension..

[15] Mosteller RD (1987). Simplified calculation of body surface area. N Engl J Med..

[16] Lang RM, Badano LP, Mor-Avi V, Afilalo J, Armstrong A, Ernande L (2015). Recommendations for cardiac chamber quantification by echocardiography in adults: an update from the American Society of Echocardiography and the European Association of Cardiovascular Imaging. European Heart Journal-Cardiovascular Imaging..

[17] Pignoli P, Tremoli E, Poli A, Oreste P, Paoletti R (1986). Intimal plus medial thickness of the arterial wall: a direct measurement with ultrasound imaging. circulation..

[18] WOCN CPWS. Ankle brachial index: quick reference guide for clinicians. J Wound Ostomy Continence Nurs 2012;39(2 Suppl):S21. 10.1097/WON.0b013e3182478dde 22415168

[19] Kim E, Wattanakit K, Gornik HL (2012). Using the ankle-brachial index to diagnose peripheral artery disease and assess cardiovascular risk. Cleve Clin J Med.

[20] Hansen TW, Jeppesen J, Rasmussen S, Ibsen H, Torp-Pedersen C (2005). Ambulatory blood pressure and mortality. Hypertension..

[21] de Luca N, Asmar RG, London GM (2004). O'rourke MF, Safar ME. Selective reduction of cardiac mass and central blood pressure on low-dose combination perindopril/indapamide in hypertensive subjects. J Hypertens.

[22] Roman MJ, Devereux RB, Kizer JR, Lee ET, Galloway JM, Ali T (2007). Central pressure more strongly relates to vascular disease and outcome than does brachial pressure. Hypertension..

[23] Spronck B, Heusinkveld MH, Vanmolkot FH, Op’t Roodt J, Hermeling E, Delhaas T (2015). Pressure-dependence of arterial stiffness: potential clinical implications. J Hypertens.

[24] Wittke E, Fuchs SC, Fuchs FD, Moreira LB, Ferlin E, Cichelero FT (2010). Association between different measurements of blood pressure variability by ABP monitoring and ankle-brachial index. BMC cardiovascular disorders..

[25] Upadhyay A, Hwang S-J, Mitchell GF, Vasan RS, Vita JA, Stantchev PI (2009). Arterial stiffness in mild-to-moderate CKD. Journal of the American Society of Nephrology..

[26] Karras Alexandre, Haymann Jean-Philippe, Bozec Erwan, Metzger Marie, Jacquot Christian, Maruani Gerard, Houillier Pascal, Froissart Marc, Stengel Bénédicte, Guardiola Philippe, Laurent Stéphane, Boutouyrie Pierre, Briet Marie (2012). Large Artery Stiffening and Remodeling Are Independently Associated With All-Cause Mortality and Cardiovascular Events in Chronic Kidney Disease. Hypertension.

